# Retrograde entry portal for femoral interlocking nailing in femoral nonunion after plate failure: a prospective comparative study with antergrade portal

**DOI:** 10.1007/s10195-016-0416-9

**Published:** 2016-07-20

**Authors:** Yasser Assaghir

**Affiliations:** Orthopaedic Department, Sohag Faculty of Medicine, Sohag University, Sohâg, 82425 Egypt

**Keywords:** Failed plate fixation, Interlocking nail femur, Retrograde portal of entry, Antegrade nailing, Femoral nonunion

## Abstract

The piriformis fossa is the ideal portal of entry for antegrade interlocking nailing. Localizing this portal can be difficult and its eccentricity leads to complications. This prospective comparative study was designed to compare an innovative way to obtain the ideal portal from inside the medullary canal in cases of plate failure and compare it to the classic antegrade portal. It included 41 cases (19 antegrade and 22 retrograde). The retrograde portal was significantly better in terms of entry time, radiation time, blood-loss, and wound length. The proper portal was rapidly and easily achieved in all retrograde cases without complications; while four in antegrade cases had complications. Minimum follow-up was 2 years.

*Level of evidence* III.

## Introduction

There are two portals of entry for antegrade femoral interlocking nailing; they are the greater trochanter and the piriformis fossa. The trochanteric tip should be reserved for nails with a proximal bend specially designed for the trochanteric portal [1]. The piriformis starting point appears to be the best, as the piriformis fossa tends to align with the longitudinal axis of the medullary canal [2]. Obtaining this ideal point can be technically difficult especially in obese patients, requires long radiation exposure, and its eccentricity can have serious consequences, specially its anterior shift [1–3]. We hypothesized we can provide an alternative technique surgery by making use of the open nature of surgery for plate failure, which must be removed, and obtain the ideal portal from inside the proximal fragment.

## Materials and methods

This study included patients with femur nonunion between 2010 and 2013. The inclusion and exclusion criteria are shown on Table 1. Forty-four cases were eligible; three were lost for follow-up; and 41 were categorized into two groups; antegrade 19 cases, and retrograde 22 cases. The inclusion into either group was based on the sequence of admission number. The demographics of the patients, nonunion, and fracture demographics are shown in Tables 2 and 3.

**Table 1:** Inclusion and exclusion criteria

Inclusion criteria	Exclusion criteria
1. Nonunion after plating	1. Septic nonunion
2. Treatment with reamed interlocking nailing	2. Plate failure after pathological fractures
3. Two years minimum follow-up

**Table 2:** The demographics of patients and nonunion are shown

Parameter	Group		Mean ± SD	*P* value
Age	Antergrade (*n* = 19)		37.1 ± 11.5	0.516
	Retrograde (*n* = 22)		38.1 ± 10.8	
Sex	Antergrade	Male	11 (57.9 %)	0.479
		Female	8 (42.1 %)	
	Antergrade	Male	12 (54.5 %)	
		Female	10 (45.5 %)	
Weight (kg)	Antergrade		84.2 ± 5.5	0.105
	Retrograde		79.1 ± 6.6	
Side	Antergrade	Right	11 (57.9 %)	0.163
		Left	8 (42.1 %)	
	Retrograde	Right	9 (40.9 %)	
		Left	13 (59.1 %)	
Level	Antergrade	Upper third	5 (26.3 %)	0.096
		Middle third	13 (68.4 %)	
		Lower third	1 (5.3 %)	
	Retrograde	Upper third	0	
		Middle third	20 (90.9 %)	
		Lower third	2 (9.1 %)	
Comminution	Antergrade	Comminuted	10 (52.6 %)	0.314
		Non-comminuted	9 (47.4 %)	
	Retrograde	Comminuted	9 (40.9 %)	
		Non-comminuted	13 (59.1 %)	
Shape	Antegrade	Transverse	8 (42.1 %)	0.254
		Oblique	8 (42.1 %)	
		Spiral	3 (15.8 %)	
	Retrograde	Transverse	5 (22.7 %)	
		Oblique	7 (31.8 %)	
		Spiral	10 (45.5 %)	
Time to plate failure	Antergrade	12.0 ± 5.2 (4–26) weeks		0.813
	Antergrade	11.3 ± 4.7 (4–24) weeks

**Table 3:** The frequency of fractures according to OTA classification

OTA type	Antegrade	Retrograde
32-A1.1	1	2
32-A1.2	2	2
32-A1.3	0	0
32-A2.1	1	0
32-A2.2	2	3
32-A2.3	2	2
32-A3.1	1	0
32-A3.2	4	6
32-A3.3	1	2
32-B1.1	0	0
32-B1.2	0	0
32-B2.1	2	1
32-B2.2	3	5
32-B2.3	0	4

*P* value between both groups was 0.249

### Operative technique

**Fig. 1:**
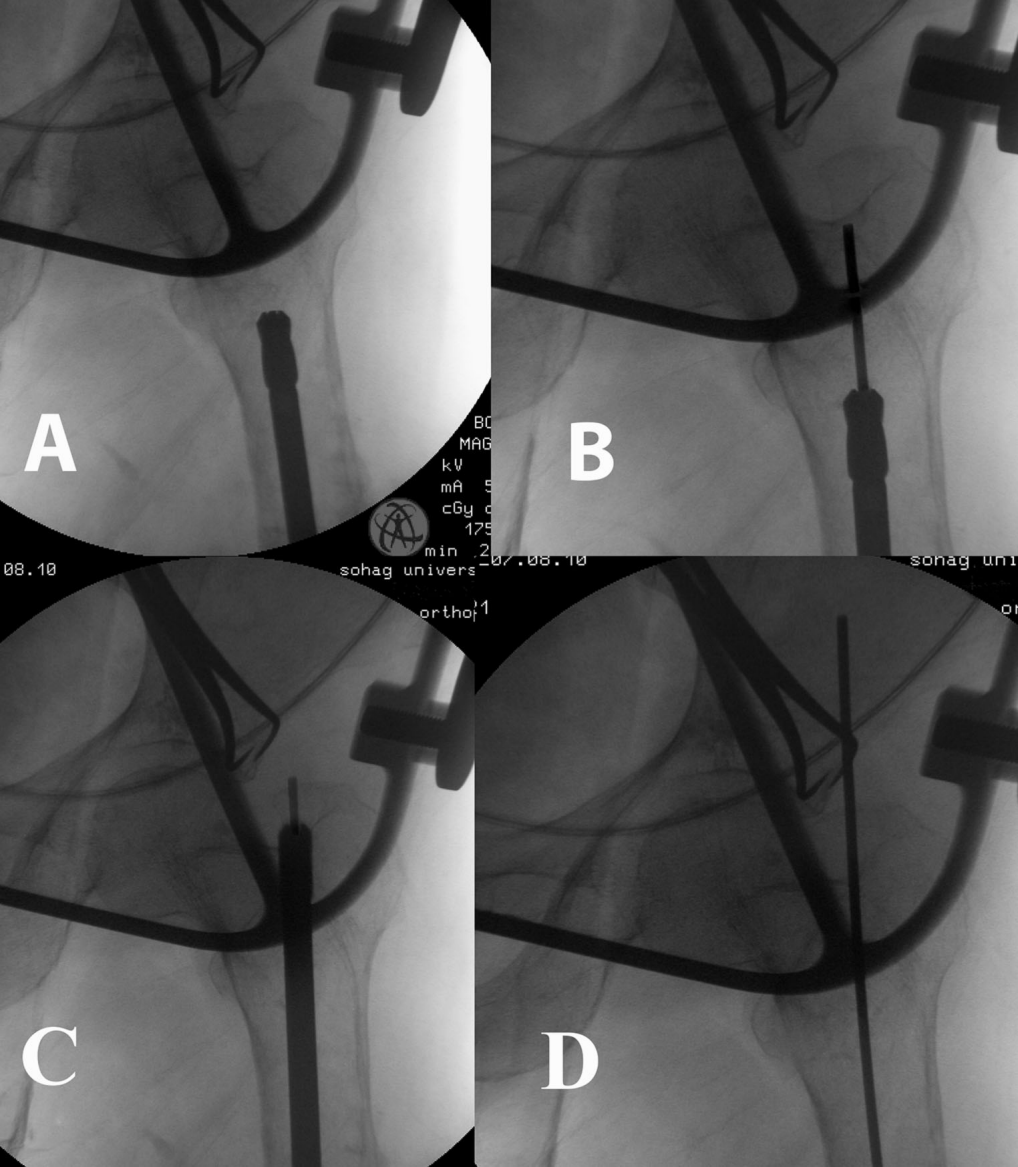
**a** The introduction of the reamer up the medullary canal. **b** The reamer guide-rod inserted through the reamer. **c** Perforation of the piriformis fossa from within the medullary canal. **d** The reamer is removed and the reamer guide-rod is exiting from the portal

**Fig. 2:**
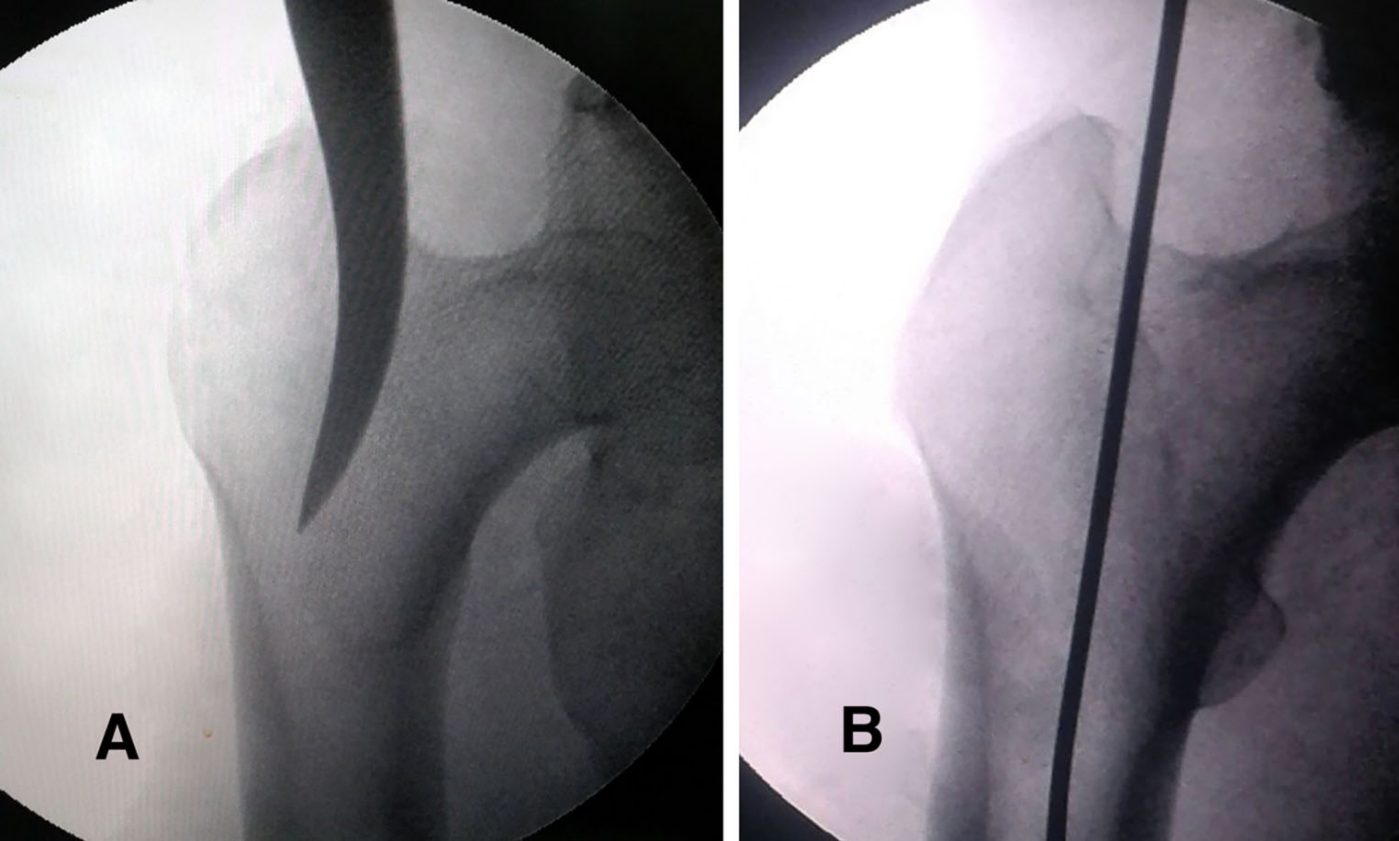
**a** Perforating the piriformis fossa with an awl. **b** Introducing the reamer guide-rod into the medullary canal in antegrade cases

Retrograde portal: hardware was removed. The AO 9 mm end-cutting reamer was introduced from the refreshed nonunion site over the reamer guide rod. After perforating the piriformis fossa, the reamer guide rod was received through a mini proximal wound (Fig. 1). The antegrade portal was done using the classic technique (Fig. 2). We used a Russell–Taylor first generation nail in all cases.

We calculated blood loss during the whole surgery, entry portal time, radiation time, and proximal incision length. Postoperative plain X-rays were requested to assess nail, locking screws, reduction, and centricity of the nail portal.

### Statistical analysis

Data was shown in the form of mean ± SD and range. Paired sample *t* test, one-way ANOVA, and Pearson test were conducted to detect significant differences between groups. All statistical analyses were done using the SPSS program (SPSS 15.0, SPSS Inc., IL, USA).

## Results

**Fig. 3:**
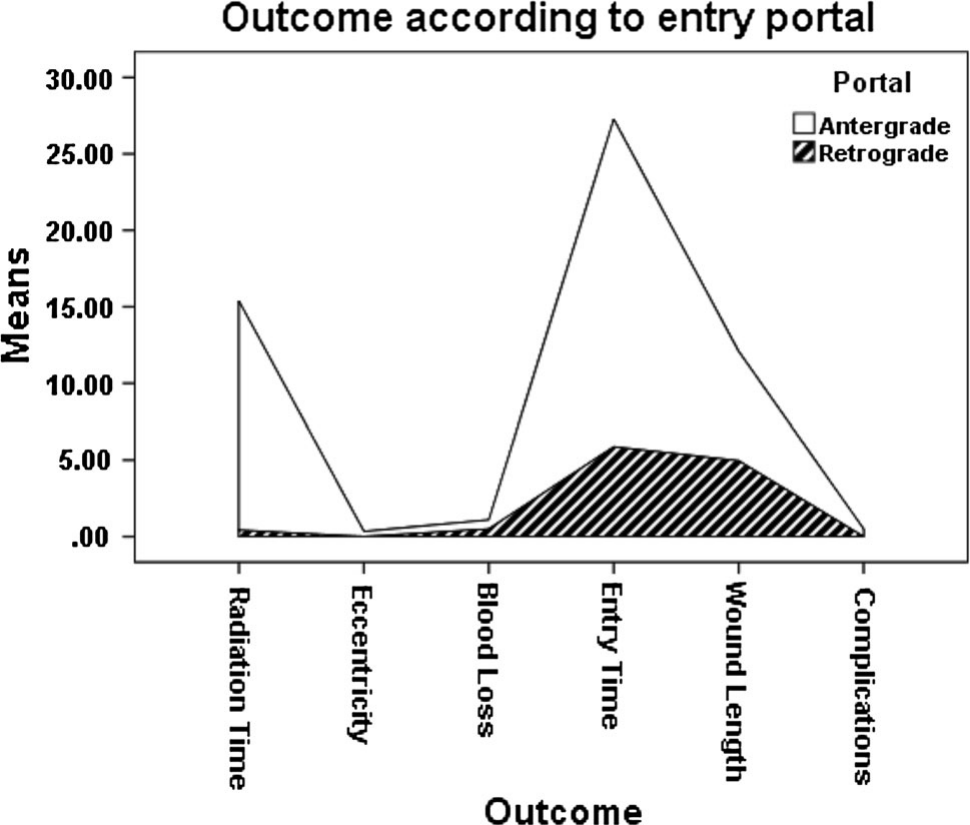
The final comparative outcome in both groups is plotted

Table 4 and Fig. 3 show the final outcome in both groups.

**Table 4:** Results with comparison between the two groups

Parameter	Mean	SD	Minimum	Maximum	*P* value
Eccentricity					
Antergrade (*n* = 19)	0.4	1.0	0.0	4.0	0.069
Retrograde (*n* = 22)	0.0	0.0	0.0	0.0	
Radiation					
Antergrade	2.7	0.8	1.8	5.6	0.000
Retrograde	0.4	0.1	0.30	0.70	
Blood loss					
Antergrade	0.58	0.10	0.45	0.85	0.015
Retrograde	0.50	0.12	0.40	0.80	
Entry time					
Antergrade	17.2	6.9	10.0	35.0	0.000
Retrograde	5.3	1.2	3.0	8.0	
Wound length					
Antergrade	7.6	1.4	5.0	10.0	0.000
Retrograde	5.0	0.61	4.0	6.0	
Sequence					
Antergrade	0.50	1.1	0.0	4.0	0.055
Retrograde	0.0	0.0	0.0	0.0

There were significant differences in favor of retrograde portal in terms of entry time, in radiation time, in blood-loss, and proximal wound length.

**Fig. 4:**
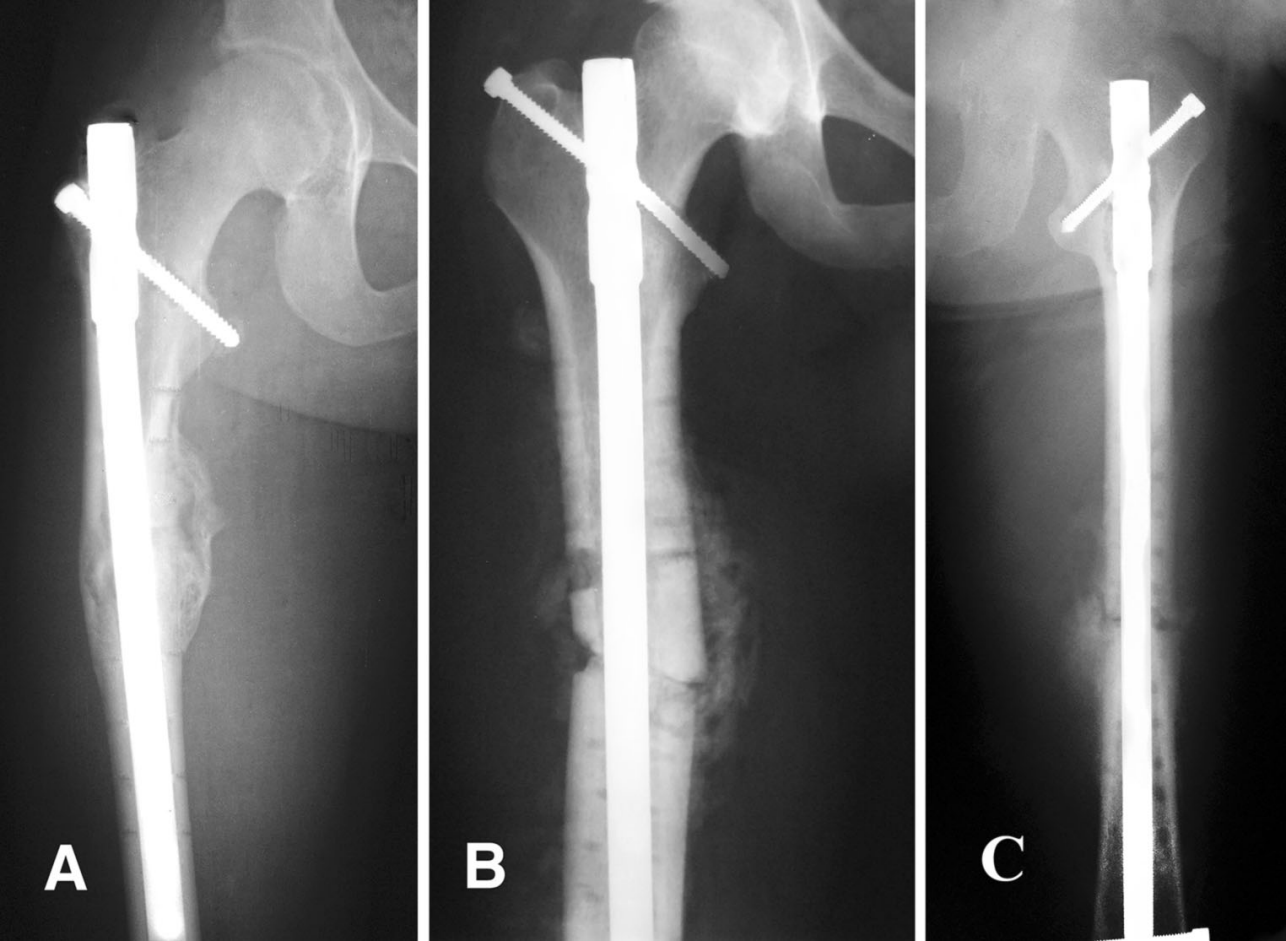
Two examples of eccentric portal of entry in antegrade cases: **a** varus angulation at nonunion site due to too lateral portal of entry into the greater trochanter. **b** Valgus angulation at nonunion site due to too medial portal of entry. While **c**, the proper portal of entry, was obtained using the retrograde technique with centralization of the nail inside the medullary canal

Proper centric entry was achieved in all cases of retrograde portal and 15 cases of antegrade portal with three cases having lateral shift of portal; and one medial shift (Fig. 4). There was a significant correlation between frontal angulation and the presence of comminuted fractures, as well eccentric portal of entry. Table 5 shows the factors causing varus/valgus angulation; Table 6 shows their statistical significance.

**Table 5:** Demographics of cases of frontal angulation

Eccentricity	Level	Shape	OTA type	Comminution	Frontal angulation
Medial	Middle third	Oblique	32-A3.2	Comminuted	5.00
Lateral	Upper third	Oblique	32-B1.1	Comminuted	6.00
Lateral	Middle third	Spiral	32-A1.2	Non-comminuted	5.00
Lateral	Middle third	Transverse	32-A3.3	Comminuted	4.00

**Table 6:** Significance of factors involved in development of frontal angulation

Dependent variable	Sig.
Eccentricity	0.014
Level	0.424
Shape	0.194
OTA type	0.360
Comminution	0.048

The Pearson test detected significant correlations between the type of portal and entry time, radiation time, blood-loss, and wound length (Table 7).

**Table 7:** Pearson test

Parameter	Pearson correlation value	Sig. (two-tailed)
Radiation	−0.886	0.000
Eccentricity	−0.332	0.034
Blood loss	−0.379	0.015
Entry time	−0.782	0.000
Wound length	−0.777	0.000
Sequence	−0.349	0.025

### Complications

Four cases in the antegrade portal group had eccentric portal: three with lateral shift had a varus angulation (5°–7°) at the nonunion site; and one medial shift had a valgus of 5° angulation.

## Discussion

Accomplishing closed reduction and locating the entry portal for nail insertion are the two most important steps in femoral interlocking nailing procedures [2]. The entry point has significant consequences for the ease of insertion and the strength of fixation [3].

There are two entry portals for antegrade femoral interlocking nailing: the piriformis fossa and the trochanteric portal [4]. A piriformis entry (ideally just posterior to its center) appears to be the best, as it tends to align with the longitudinal axis of the medulla [2, 5, 6]. Its disadvantages are the relative technical difficulty compared with retrograde and trochanteric portals [7]. The trochanteric portal is the tip of greater trochanter and its use is limited to nails with a proximal bend for paediatric fractures [8]. The trochanteric-tip portal has a much greater potential of iatrogenic proximal femoral fractures during nail insertion [9]. Both portals do not affect the perfusion of the femoral head [10].

This study tried to test the use of a novel method of obtaining the proper piriformis portal from inside the medulla in a specific situation of femoral nonunion after failed plating, and compared it to the classic antegrade portal.

The weakness of this study is the weak quasi-randomization, and the small number of cases. The strengths are the prospective randomized nature, the numerous statistically significant differences between groups, and the success of obtaining the centric entry in all cases.

Compared to the classic antegrade portal, it had a 69.1 % shorter portal time; 13 % less blood loss; 85 % reduction in fluoroscopy time; and 34 % reduction of entry wound length. The ideal portal was obtained in all retrograde cases with no angulation, eccentricity, or comminution.

The new technique is easier to perform with numerous advantages. It provides a better alternative surgical technique than the classic antegrade portal in femoral nonunion after plate failure or similar conditions requiring open nailing.

## References

[1] Ricci WM, Gallagher B, Haidukewych GJ (2009). Intramedullary nailing of femoral shaft fractures: current concepts. J Am Acad Orthop Surg.

[2] Winquist RA (1993). Locked femoral nailing. J Am Acad Orthop Surg.

[3] Linke B, Ansari Moein C, Bosl O, Verhofstad MH, van der Werken C, Schwieger K, Ito K (2008). Lateral insertion points in antegrade femoral nailing and their influence on femoral bone strains. J Orthop Trauma.

[4] Charopoulos I, Giannoudis PV (2009). Ideal entry point in antegrade femoral nailing: controversies and innovations. Injury.

[5] Johnson KD, Tencer AF, Sherman MC (1987). Biomechanical factors affecting fracture stability and femoral bursting in closed intramedullary nailing of femoral shaft fractures, with illustrative case presentations. J Orthop Trauma.

[6] Gausepohl T, Pennig D, Koebke J, Harnoss S (2002). Antegrade femoral nailing: an anatomical determination of the correct entry point. Injury.

[7] Tucker MC, Schwappach JR, Leighton RK, Coupe K, Ricci WM (2007). Results of femoral intramedullary nailing in patients who are obese versus those who are not obese: a prospective multicenter comparison study. J Orthop Trauma.

[8] Prasarn ML, Cattaneo MD, Achor T, Ahn J, Klinger CE, Helfet DL, Lorich DG (2010). The effect of entry point on malalignment and iatrogenic fracture with the Synthes lateral entry femoral nail. J Orthop Trauma.

[9] Tupis TM, Altman GT, Altman DT, Cook HA, Miller MC (2012). Femoral bone strains during antegrade nailing: a comparison of two entry points with identical nails using finite element analysis. Clin Biomech (Bristol Avon).

[10] Schottel PC, Hinds RM, Lazaro LE, Klinger C, Ni A, Dyke JP, Helfet DL, Lorich DG (2015). The effect of antegrade femoral nailing on femoral head perfusion: a comparison of piriformis fossa and trochanteric entry points. Arch Orthop Trauma Surg.

